# Myalgic encephalomyelitis or chronic fatigue syndrome: how could the illness develop?

**DOI:** 10.1007/s11011-019-0388-6

**Published:** 2019-02-13

**Authors:** Gerwyn Morris, Michael Maes, Michael Berk, Basant K. Puri

**Affiliations:** 1 IMPACT Strategic Research Centre, School of Medicine, Barwon Health, Deakin University, Geelong, Victoria Australia; 2 Department of Psychiatry, Faculty of Medicine, Chulalongkorn University, Bangkok, Thailand; 3 Department of Psychiatry, Royal Melbourne Hospital, University of Melbourne, Parkville, Victoria Australia; 4 Florey Institute for Neuroscience and Mental Health, University of Melbourne, Parkville, Victoria Australia; 5 Orygen, The National Centre of Excellence in Youth Mental Health, Parkville, Victoria Australia; 6 Department of Medicine, Imperial College London, Hammersmith Hospital, London, England W12 0HS UK

**Keywords:** Chronic fatigue syndrome, Myalgic encephalomyelitis, Endotoxin tolerance, Inflammation, Mitochondria, Oxidative and nitrosative stress

## Abstract

A model of the development and progression of chronic fatigue syndrome (myalgic encephalomyelitis), the aetiology of which is currently unknown, is put forward, starting with a consideration of the post-infection role of damage-associated molecular patterns and the development of chronic inflammatory, oxidative and nitrosative stress in genetically predisposed individuals. The consequences are detailed, including the role of increased intestinal permeability and the translocation of commensal antigens into the circulation, and the development of dysautonomia, neuroinflammation, and neurocognitive and neuroimaging abnormalities. Increasing levels of such stress and the switch to immune and metabolic downregulation are detailed next in relation to the advent of hypernitrosylation, impaired mitochondrial performance, immune suppression, cellular hibernation, endotoxin tolerance and sirtuin 1 activation. The role of chronic stress and the development of endotoxin tolerance via indoleamine 2,3-dioxygenase upregulation and the characteristics of neutrophils, monocytes, macrophages and T cells, including regulatory T cells, in endotoxin tolerance are detailed next. Finally, it is shown how the immune and metabolic abnormalities of chronic fatigue syndrome can be explained by endotoxin tolerance, thus completing the model.

## Introduction

Research into the cause and treatment of myalgic encephalomyelitis (ME), also known as chronic fatigue syndrome (CFS), has involved the use of 20 different case definitions in common use (Brurberg et al. 2014). The widest definition of CFS is favoured in the UK and only mandates the presence of idiopathic fatigue of variable severity (Sharpe et al. 1991), while the narrowest definition favoured by investigating physicians in the USA mandates the presence of severe incapacitating fatigue, pain, compromised sleep, neurocognitive disability symptoms consistent with autonomic dysfunction and a worsening of global symptoms following even trivial increases in activity (Carruthers et al. 2011). This is an important issue as the use of narrow selection criteria identify patients with far higher levels of physical and cognitive disability than the use of wider criteria (Jason et al. 2012, 2015a, 2016) and criteria variance has been identified as the main factor accounting for the lack of replicated data which has impeded progress in this field (Jason et al. 2015a, b) (reviewed (Morris and Maes 2013a)). Using published criteria, the prevalence of ME/CFS is relatively high, at between 0.2 and 6.4%; together with a low level of employment, reported to be between 27 and 41%, it is clear that this disorder poses a high financial burden on patients and society (Johnston et al. 2013; Rimbaut et al. 2016). It also carries a high cost in terms of symptomology; compared with patients suffering from multiple sclerosis (MS), CFS patients have been reported to suffer from higher levels of symptom severity, higher levels of depression and kinesiophobia, and lower quality of life, lower maximum voluntary muscle contraction and muscle recovery, and lower cognitive performance (Meeus et al. 2016). In spite of this disease burden, research into ME/CFS is relatively low. Indeed, it has been calculated that, in the USA, if the federal research funding for ME/CFS were to take disease burden into account, then, by comparison with the funding pattern for other diseases, the funding for ME/CFS research would need to be increased by at least a factor of 25 (Dimmock et al. 2016).

However, there is a large and accumulating body of evidence reporting the existence of a wide range of biological abnormalities in patients afforded a diagnosis of CFS according to current international consensus criteria (Fukuda et al. 1994), most notably in the neuroendocrine, autonomic, neurological, bioenergetic, redox and immunological domains (Morris and Maes 2013b, c). It is germane to note that, in the USA, the National Institute of Neurological Disorders and Stroke have developed the common data elements for clinical research in mitochondrial disease project ‘to provide clinical researchers with tools to improve data quality and allow for harmonization of data collected in different research studies’ (Karaa et al. 2017, 2018). Common data elements for ME/CFS are being developed along the lines of the following 11 domains: baseline/covariate; fatigue; post-exertional malaise; sleep; pain; neurologic/cognitive/CNS imaging; autonomic; neuroendocrine; immune; quality of life/functional status/activity; and biomarkers. Eleven corresponding subgroups first met in 2017; a related publication will be written in due course.

Early results indicate that increased production of intracellular nuclear-factor 6B and cyclo-oxygenase-2 (COX-2) may be key phenomena in CFS indicating activation of immune-inflammatory pathways in that illness (Maes et al. 2006). In addition, increased production of inducible nitric oxide (NO) synthase (iNOS) coupled with increased IgM responses to NO-adducts such as NO-tryptophan indicate increased nitrosylation of proteins (Maes et al. 2006).

Numerous research teams have reported the presence of an activated but dysregulated immune system with elevated pro-inflammatory cytokines (PICs), T cell anergy, natural killer (NK) cell dysfunction, and Th1, Th2 and, possibly, Th17 lymphocyte biases being repeatedly reported (Brenu et al. 2011; Hornig et al. 2015, 2017; Maes et al. 2012a, b; Milrad et al. 2017; Montoya et al. 2017; Peterson et al. 2015; Russell et al. 2016). There is also evidence of a longitudinal shift in the immune profiles of patients, with an inflammatory phenotype seen in early disease giving way to an anti-inflammatory or immunosuppressed phenotype, indicating activation of the compensatory anti-inflammatory reflex system (Morris and Maes 2013a, b, c) and somewhat reminiscent of the profile seen in endotoxin tolerance in patients who have been ill for many years or even decades (Hornig et al. 2015; Russell et al. 2016). Readers interested in a detailed review of these data are referred to reviews by (Morris and Maes 2013b; Morris et al. 2015a). It should be stressed, however, that there is no evidence of any immune abnormalities in participants afforded a diagnosis of CFS based on any diagnostic schema other than the Fukuda or Canadian criteria (Blundell et al. 2015).

Reported markers of chronic oxidative and nitrosative stress (ONS) include elevated levels of reactive oxygen species (ROS) and reactive nitrogen species (RNS), depleted levels of reduced glutathione (GSH), elevated inducible nitric oxide synthase (iNOS) and oxidatively modified proteins and highly reactive metabolites of lipid peroxidation such as 4-hydroxynonenal and malondialdehyde together with the presence of autoantibodies directed at neoepitopes and the presence of damage associated molecular patterns (DAMPs) (Fulle et al. 2000, 2007; Gerwyn and Maes 2017; Maes 2013; Morris et al. 2015a; Morris and Maes 2013b, c; Rutherford et al. 2016).

Importantly, several research teams have reported that levels of oxidative stress in the muscles of exercising CFS patients are higher than in age- and sex-matched controls and that protective heat shock protein (HSP) responses are impaired (Jammes et al. 2005, 2009, 2011, 2012; Thambirajah et al. 2008). The existence of oxidative stress in individuals afforded a diagnosis of CFS using schemata other than the Canadian or Fukuda criteria is currently uncertain as a literature search failed to uncover any published research investigating this matter.

Chronic ONS is an acknowledged cause of mitochondrial dysfunction (Morris et al. 2017c, d) and hence the fact that impaired synthesis of adenosine triphosphate (ATP), impaired oxidative phosphorylation and damaged or morphologically abnormal mitochondria in striated muscle and peripheral mononuclear blood cells (PMBCs) of patients have all been extensively reported is unsurprising (Morris and Berk 2015; Morris and Maes 2013b; Naviaux et al. 2016; Tomas et al. 2017).

Inflammation, oxidative stress and mitochondrial dysfunction are also recognised drivers of neuroendocrine and autonomic system dysfunction (Kanjwal et al. 2010; Masson et al. 2015; Schultz 2009; Ulleryd et al. 2017), hence the existence of neuroendocrine abnormalities (reviewed (Morris et al. 2017a; Tomas et al. 2013)) and dysautonomia (Lewis et al. 2013; Naschitz et al. 2004, 2006; Newton et al. 2007; Van Cauwenbergh et al. 2014) is to be expected. This is of importance as some 90% of patients diagnosed via the Fukuda criteria have evidence of autonomic dysfunction characterised by increased sympathetic activity, decreased parasympathetic activity and vagal nerve hypoactivity (Beaumont et al. 2012; Lewis et al. 2013; Robinson et al. 2015). The most common manifestation of dysautonomia reported in trial participants is a suppressed and unresponsive heart rate variability (HRV) both during the day and at night (Boneva et al. 2007; Burton et al. 2010; Kadota et al. 2010; Vollmer-Conna et al. 2006). These findings have been confirmed by a large meta-analysis (Martinez-Martinez et al. 2014). Once again it should be stressed that there is no evidence of any abnormal HRV values in patients afforded a diagnosis of CFS via the application of one of a plethora of alternative criteria (Bozzini 2012; Malfliet et al. 2018).

Disrupted patterns of resting state functional connectivity have been repeatedly reported in patients and appear to correlate with levels of fatigue and pain (Boissoneault et al. 2016; Gay et al. 2016; Kim et al. 2015b; Wortinger et al. 2016). The use of voxel-based morphometric analysis of structural magnetic resonance imaging (MRI) brain scans has revealed abnormalities in brain structure and regional volumes (Barnden et al. 2011, 2015; Finkelmeyer et al. 2018; Puri et al. 2012; Shan et al. 2016), including grey matter (GM) and white matter (WM) changes (de Lange et al. 2005, 2008; Finkelmeyer et al. 2018; Okada et al. 2004; Puri et al. 2012; Shan et al. 2016). These findings contrast with earlier work with low resolution MRI and manual analysis which revealed abnormalities in some patients but not others (Perrin et al. 2010) (reviewed (Morris et al. 2017b)).

Systematic studies of cerebral chemistry, using magnetic resonance spectroscopy, have shown evidence of increased regional levels of choline-containing compounds (Chaudhuri et al. 2003; Puri et al. 2002).

The existence of regional or global cerebral hypoperfusion indicative of reduced bioenergetic capacity has also been consistently reported by research teams utilising xenon-computed tomography, arterial spin labelling and high-resolution single-photon emission computed tomography (SPECT) (Biswal et al. 2011; Machale et al. 2000; Patrick Neary et al. 2008; Yoshiuchi et al. 2006). These findings have also been reported in large studies utilising older SPECT techniques either globally (Ichise et al. 1992; Schwartz et al. 1994) or regionally (Costa et al. 1995; Goldstein et al. 1995) but the results in studies with far fewer participants have been negative (Fischler et al. 1996; Peterson et al. 1994).

Unsurprisingly, given the evidence regarding neuroimaging abnormalities above, a meta-analysis of 50 studies has confirmed the existence of widespread cognitive dysfunction in patients, which are at their worst in the domains of attention, memory, reaction times, information processing and reasoning (Cockshell and Mathias 2010). These findings are also discussed in two excellent narrative reviews by (Cvejic et al. 2016; Shanks et al. 2013) and global decreases in cognitive and executive functions have recently been reported in adults and adolescents irrespective of sex (Nijhof et al. 2016; Santamarina-Perez et al. 2014). It is interesting that several research teams using functional MRI (fMRI) have noted that cognitive dysfunction worsens in patients with increased effort and/or exercise, as such findings may be of relevance from the perspective of pathogenesis and/or pathophysiology (Cook et al. 2017; DeLuca et al. 2004; Lange et al. 2005; Tanaka et al. 2006). It is also noteworthy that a recent large study primarily containing participants with CFS confirmed the results of earlier research indicating that there was scant evidence of global cognitive dysfunction in patients diagnosed via alternative criteria (Hughes et al. 2018).

Many patients complain of ‘brain fog’, which is often described as slow thinking, difficulty focusing, slow thinking, lack of concentration, confusion, forgetfulness, or, sometimes, hazy thought processes (Ocon 2013). From a more objective perspective, subjective brain fog can be described as a constellation of symptoms that include impaired cognition, loss of long- and short-term memory, and a reduced ability to concentrate and engage in multiple low-level tasks at once (Theoharides et al. 2015). It is important to note that this phenomenon is not confined to CFS patients but also characterises patients with autism spectrum disorders, coeliac disease, postural orthostatic tachycardia syndrome, as well as patients with mild cognitive impairment and a range of neuroprogressive illnesses (reviewed (Theoharides et al. 2015)). The causes of brain fog are not fully delineated but there is accumulating evidence that this symptom complex may relate to autonomic dysfunction and/or decreased regional cerebral blood flow (rCBF) in the context of ongoing neuroinflammation (Ocon 2013; Ross et al. 2013; Theoharides et al. 2015).

Several research teams have reported changes in the DNA sequences or expression of numerous genes involved in the delivery of the immune response and the regulation of metabolic and bioenergetic pathways in patients diagnosed according to the Fukuda criteria (Morris et al. 2016b). Examples of dysfunctional or abnormally expressed genes compared with matched controls include *NFKB1*, *IL6*, *IL1A*, *TNF*, *IL17A*, *IL7*, *CXCL8* (formerly *IL8*), *INFG*, *IRF3*, *TLR4*, *CD14*, *STAT5A*, *HSPA2*, *P2RX7*, *ATP5J2*, *GZMA*, *COX5B*, *DBI*, *PSMA3*, *PSMA4*, *HINT*, *ARHC*, *HLA-DQB1-AS1*, *RIPK3* and *DEFA1* (Carlo-Stella et al. 2006; Gow et al. 2009; Kerr et al. 2008a, b; Light et al. 2009, 2012, 2013; Nguyen et al. 2017; Saiki et al. 2008; Shimosako and Kerr 2014; White et al. 2012; Zhang et al. 2010). There is also evidence of abnormalities in the mitochondrial genome of patients as a research team has recently reported the presence of polymorphisms in the mitochondrial DNA (mtDNA) of their trial participants which were associated with increased symptom severity (Billing-Ross et al. 2016). These findings were reiterated in (Hanson et al. 2016). These are interesting observations as this study contained 196 patients who were recruited via criteria which mandated the existence of what many researchers view as the defining characteristic of CFS, namely an exacerbation of symptoms following even trivial increases in activity (Morris and Maes 2013a).

The observation that mtDNA polymorphisms appear to influence the severity of CFS is consistent with observations in other disease areas where such polymorphisms increase the susceptibility to the development of metabolic and neurodegenerative diseases and susceptibility to microbial infection (review (Hendrickson et al. 2008)). Polymorphisms in mtDNA also play a role in structuring the composition of the microbiota and determining the levels of IgG and IgM autoantibody production (Ma et al. 2014; Zhou et al. 2017). This may be of pathophysiological relevance in the light of data demonstrating elevated IgA and IgM responses to lipopolysaccharide (LPS)/antigens of *Gram-negative* gut commensal bacteria and gut dysbiosis in patients afforded a diagnosis of CFS via the Fukuda criteria (Maes et al. 2006; Morris et al. 2016b; Morris and Maes 2013b). Mutations in mtDNA can increase levels of inflammation and oxidative stress (I&OS) via direct effects on the innate immune system involving PIC production and NF-κB activity and hence can influence the intensity of the immune response (Imanishi et al. 2013; Ishikawa et al. 2010; Novak and Mollen 2015).

There is also evidence of abnormalities in the epigenetic regulation of gene expression in CFS patients diagnosed via narrow criteria, most notably in gene promoter methylation patterns and elevation of microRNAs (miRNAs) involved in the regulation of the immune system (Brenu et al. 2014a; de Vega et al. 2014, 2017; Petty et al. 2016; Vangeel et al. 2015, 2018). The work of de Vega and others is of particular interest as these authors also selected patients according to criteria mandating the presence of post-exertional malaise and examined global patterns of gene methylation rather than a single gene as was the case for Vangeel and colleagues (de Vega et al. 2014, 2017; Vangeel et al. 2015, 2018).

Importantly, de Vega and others reported a global hypomethylation of cytosine residues in the promoter regions of immune system-related genes consistent with a chronically activated but dysregulated immune system, and abnormal patterns of DNA methylation in genes regulating metabolic pathways and various aspects of cellular homeostasis (de Vega et al. 2014, 2017). The work of (Vangeel et al. 2015, 2018) is also of interest as the pattern of hypomethylation of the glucocorticoid receptor gene *NR3C1* 1F region suggests an activated hypothalamic-pituitary-adrenal (HPA) axis in an attempt to counter peripheral inflammation rather than a blunted HPA response reported in people diagnosed with CFS according to wider criteria (reviewed (Morris et al. 2017a)). The lack of association between childhood trauma and levels of methylation reported by these authors in studies where participants universally reported this phenomenon is also of interest as these patients were diagnosed according to the Fukuda criteria whereas studies which have reported a significant but slight association between childhood trauma and CFS involved participants recruited via alternative criteria (reviewed (Morris et al. 2017a)).

Abnormalities in miRNA levels in CFS patients diagnosed according to the Fukuda criteria may also suggest dysregulation of immune and metabolic pathways. For example, upregulated *hsa-miR-127-3p*, *hsa-miR-142-5p* and *hsa-miR-143-3p* was reported by (Brenu et al. 2014a) in an analysis of whole blood profiles and elevated expression of *hsa-miR-99b*, *hsa-miR-330*, *hsa-miR-126* and *hsa-miR-30c* was reported by (Petty et al. 2016) in an analysis involving NK cells and monocytes. *miR-99b* upregulation is involved in immune downregulation in macrophages and DCs by reducing levels of IL-6, IL-12 and IL-1β upregulated in response to an infection (Singh et al. 2013; Zheng et al. 2015). *miR-127-5p* upregulation exerts an immunosuppressive effect by inhibiting the phosphorylation and subsequent translocation of p65 into the nucleus leading to the inhibition of NF-κB signalling and the downregulation of c-Jun N-terminal kinase (JNK)/p38 and reduced levels of PICs (Huan et al. 2016; Park et al. 2013). *miR-30* also acts to suppress the immune response by inhibiting the toll-like receptor (TLR)/myeloid differentiation primary response 88 (MyD88) signalling pathway (Wu et al. 2017). Upregulation of *miR-143* and *miR-142-5* also signals a downregulated immune response as the production both molecules is increased following transforming growth factor beta 1 (TGF-β1) activation and plays positive and inhibitory roles in signalling pathways instigated by this cytokine (Cheng et al. 2014b; Long and Miano 2011; Ma et al. 2016; Yu et al. 2017). miR-126 is an effector of TGF-β1 signalling and regulates the activity of the PI3K/Akt signalling pathway (Guo et al. 2008, 2016). This molecule also regulates multiple aspects of the immune response in macrophages and monocytes, plays a major role in the preconditioning of the immune system against future infections with the same pathogen and activates mechanistic (or mammalian) target of rapamycin kinase (mTOR) and glycogen synthase kinase 3 (GSK-3) (Ye et al. 2013) (reviewed (Ferretti and La Cava 2014; Bai et al. 2014)). In addition, miR-126 regulates cytosolic TLR signalling and modulates the duration and intensity of such signalling in DCs (Rogers and Herzog 2014). Finally, miR-330 also regulates the activity of the PI3K/Akt signalling pathway and also exerts an inhibitory effect on T cell proliferation and NK cell activation (Grigoryev et al. 2011; Kim et al. 2015a; Petty et al. 2016).

Several pathogens have been associated with the development of CFS and evidence of chronic virus infections have been repeatedly reported in the gastrointestinal (GI) tract (Chia et al. 2010; Chia and Chia 2008), serum (Clements et al. 1995) and muscle (Cunningham et al. 1991; Gow and Behan 1991; Lane et al. 2003). Pertinently, these latter findings were not replicated in patients diagnosed according to alternative criteria (Swanink et al. 1994). Several authors have reported the presence of activated Epstein-Barr virus, human herpesvirus 6, cytomegalovirus and parvovirus B19, but these findings are not universal even in patients diagnosed according to narrow criteria (Morris and Maes 2013b) reviewed (Morris et al. 2016d)). Hence it is difficult to conclude that persistent or chronic infections are at the root of CFS/ME/SEID, but of course they could be in some patients. However, many patients have a history of a severe infection before the development of their symptoms (Gow et al. 2009; Hickie et al. 2006; Stormorken et al. 2015; White 2007; Zhang et al. 2010).

In this context it is noteworthy that the intensity of the immune response is not determined solely by the virulence or otherwise of an invading pathogen but by genetic and epigenetic variation in immune response genes (Bronkhorst et al. 2013; Morandini et al. 2016; Rautanen et al. 2015; Smelaya et al. 2016). Epigenetic variation in immune response genes also plays a major role in determining the development of DAMPs in an individual post-infection (reviewed (Morandini et al. 2016)). This may be pertinent from the perspective of the aetiology of CFS, as the production or presence of these molecules can at least in some circumstances ‘convert’ an acute pathogenic infection into a state of escalating chronic systemic inflammation, and hence this mechanism could conceivably underpin the development of chronic symptoms in CFS patients diagnosed according to the Fukuda criteria (Lucas and Maes 2013; Lucas et al. 2015). However, could this relatively simple concept explain all the observations relating to CFS reviewed above? Accordingly, this paper aims to answer this question in the context of developing an explanatory model of illness development and progression, commencing with a proposed mechanism explaining the development of chronic systemic inflammation, oxidative and nitrosative stress (I&ONS) following a pathogen invasion in genetically predisposed individuals.

## Acute infection and the development of chronic I&ONS: The role of DAMPs

The relationship between polymorphisms in immunity-related genes and the intensity of the immune response, irrespective of pathogen virulence, has been repeatedly demonstrated (Cvejic et al. 2014; Helbig et al. 2005; Piraino et al. 2012; Vollmer-Conna et al. 2008). Equally, the relationship between an exaggerated immune response and increased production of DAMPs stemming from cellular stress and tissue damage is well documented (Fichna et al. 2016; Kakihana et al. 2016; Rittirsch et al. 2008; Wiersinga et al. 2014).

Mechanistically, the genesis of DAMPs following TLR or nucleotide-binding oligomerisation (NOD)-like receptor activation by a range of pathogens in the context of a hyper-responsive immune system involves prolonged or excessive activation of NFκB and other transcription factors such as nuclear factor of activated T cells and activated protein-1. This leads to upregulation of macrophage and monocyte PICs and activation of T and B lymphocytes (Lucas et al. 2015; Morris et al. 2015a). Abnormally elevated PIC production in turn leads to elevated upregulation of iNOS and nicotinamide adenine dinucleotide phosphate (NADPH) oxidase, leading to the production of superoxide, nitric oxide and peroxynitrite, leading to further upregulation of NF-κB and hence further increases in PICs, ROS and RNS levels (Morris et al. 2015a; Morris and Maes 2014). This bidirectional self-amplifying association between the development of chronic systemic inflammation and chronic ONS is sometimes described as an ‘autotoxic loop’ (Ortiz et al. 2013; Reuter et al. 2010). Excessive levels of ROS and RNS can lead to damage to proteins, lipids and DNA and the formation of oxidative specific epitopes and products of lipid peroxidation, which function as DAMPs capable of activating TLRs on cell membranes and cytosolic pathogen recognition receptors (PRRs) (Bowie 2013; Leibundgut et al. 2013; Miller et al. 2011; Uchida 2013).

Importantly, several research teams have reported the presence of such DAMPs in CFS patients (Brkic et al. 2010; Maes et al. 2011; Maes and Leunis 2014; Richards et al. 2007; Tomic et al. 2012; Wang et al. 2014). In addition, there is accumulating evidence indicating that an environment of chronic ONS leads to release of mitochondrial components into the cytosol which also have the capacity to activate cytosolic PRRs and logically are categorised as mitochondrial DAMPs (reviewed (Nakahira et al. 2015)). There are a range of molecular entities which fall into this category other than mtDNA and one such species is cardiolipin (Wenceslau et al. 2014). This is of importance from the perspective of this paper as immunogenic cardiolipin has been repeatedly detected in CFS patients diagnosed according to internationally agreed criteria (Hokama et al. 2008, 2009). The work of Hokama and others would appear to be especially noteworthy as the study contained 320 participants satisfying the requirements of the Fukuda criteria (Hokama et al. 2008).

Research investigating the role of DAMPs in human diseases is advancing apace and the categorisation of these molecules (Jammes et al. 2009) as uniquely proinflammatory entities is changing; one such change which may be relevant to the pathogenesis of CFS is the realisation that HSPs, once thought to be exclusively proinflammatory, have a key anti-inflammatory function and play a crucial role in restraining the intensity and/or duration of the immune response (van Eden et al. 2012) (reviewed (van Eden et al. 2017)). This is pertinent given that HSP production appears to be deficient in CFS patients, and the HSPs produced appear to be dysfunctional, which could potentially provide another mechanism underpinning a prolonged and/or exaggerated immune response to pathogen invasion and other sources of inflammation such as stress, medical comorbidity and lifestyle factors in such patients (Elfaitouri et al. 2013; Jammes et al. 2005, 2009, 2011, 2012).

Chronic engagement of TLRs by DAMPs leads to the development of a positive feedback loop, whereby increasing tissue damage caused by elevated PICs, ROS and RNS perpetuates and escalates pro-inflammatory responses, leading to a state of chronic inflammation, ONS, mitochondrial dysfunction and glial cell activation (Drexler and Foxwell 2010; Goh and Midwood 2012; Morris and Berk 2015; Piccinini and Midwood 2010). Unsurprisingly, chronic engagement of TLRs, NOD-like receptors and retinoid acid-inducible gene I (RIG-I)-like receptors is implicated in the pathogenesis and pathophysiology of systemic lupus erythematosus (SLE), rheumatoid arthritis and MS (review (Drexler and Foxwell 2010; Goh and Midwood 2012; Piccinini and Midwood 2010)). Pertinently, the presence of DAMPs can also lead to chronic activation of the inflammasome (Anders and Schaefer 2014), which is also implicated in the development of neuro-inflammation and abnormal central nervous system (CNS) signalling characteristic of neurodegenerative and neurodevelopmental disorders (Singhal et al. 2014; Tan et al. 2013).

## Consequences of chronic systemic I&ONS

### Increased intestinal permeability and translocation of commensal antigens into the circulation

Chronically elevated I&ONS in the guise of increased levels of ROS, RNS and PICs induces marked increases in intestinal epithelial barrier permeability (Al-Sadi et al. 2009; Banan et al. 2003; Lee 2015; Tian et al. 2017), ultimately leading to translocation of Gram-negative bacterial LPS and a range of other commensal antigens such as peptidoglycan and flagellin, from the gut lumen into the intestinal mucosa (Lucas et al. 2015; Morris et al. 2016b). Such events lead to the creation of a self-amplifying pattern of localised and then systemic inflammation via several different routes (Delzenne and Cani 2011; Zhang and Zhang 2013).

In the first instance, the presence of LPS in the colon exacerbates inflammation in the intestine and depletes levels of regulatory T cells (Tregs), resulting in increased PIC levels (Im et al. 2012). In addition, increased an concentration of colonic LPS provokes further increases in epithelial tight junction permeability by stimulating the increased synthesis and release of the chemokine IL-8 by intestinal epithelial cells and upregulation of TLR4 on the surface of enterocytes (Angrisano et al. 2010; Guo et al. 2013). Translocated LPS also increases tight junction permeability by inducing increased enterocytic expression of *TLR4* and *CD14* and changes in the location of their respective proteins (Guo et al. 2013).

The development of gut inflammation interacts with dysbiosis of the microbiome and provokes the recruitment of proinflammatory macrophages into mucosal tissue which exacerbates localised inflammation, further increasing intestinal permeability to the point that enables the translocation of LPS, peptidoglycan and flagellin into the bloodstream (Delzenne and Cani 2011; Zhang and Zhang 2013). This latter phenomenon can have serious consequences in terms of initiating, or in this instance exacerbating, systemic inflammation via the activation of TLR4 and TLR2 on antigen-presenting cells and increasing levels of PICs, ROS and RNS (Morris et al. 2015a, b). The pathogenic consequences of LPS translocation are highlighted by replicated data demonstrating that this phenomenon is the cause of chronic systemic immune activation and I&OS seen in HIV seropositive patients in the absence of viraemia and otherwise well controlled on highly active antiretroviral therapy (Brenchley and Douek 2008; Shan and Siliciano 2014). LPS translocation into the systemic circulation is also held to be the cause of the metabolic endotoxaemia increasingly considered to be a major element in the pathogenesis of a wide range of inflammatory conditions and illnesses such as metabolic syndrome, type 2 diabetes mellitus and MS (Cani et al. 2008, 2009; Puddu et al. 2014; Riccio and Rossano 2015). From the perspective of a model of the pathogenesis and pathophysiology of ME/CFS, the important point is that the advent of LPS translocation in an environment of pre-existing I&ONS would be expected to increase the levels of all these parameters.

### Development of autonomic dysfunction

A plethora of human studies have established a causative association between indices of increased systemic inflammation, most notably elevated C-reactive protein and IL-6, and low and unresponsive HRV in a range of inflammatory and infectious illnesses such as MS, type 2 diabetes mellitus and sepsis (de Castilho et al. 2017; Stuckey and Petrella 2013; Studer et al. 2017; Tateishi et al. 2007). More specifically, translocated LPS-mediated systemic inflammation is an acknowledged cause of low and unresponsive HRV in people with metabolic endotoxaemia, which is of relevance given the existence of this phenomenon in many CFS patients (Jan et al. 2010; Lehrer et al. 2010; Morris et al. 2016b). However, the suppressive effect of LPS on HRV is not limited to its effects on systemic inflammation as there is evidence that LPS may also invoke systemic autonomic dysfunction via direct effects on TLR4 receptors on microglia in the paraventricular nucleus of the hypothalamus (Masson et al. 2015; Okun et al. 2014) and the brainstem (Ogawa et al. 2011). The effects of LPS in this instance appear to be mediated via cross-talk with angiotensin II and the ultimate effect is the activation of microglia with resultant increases in I&OS throughout the hypothalamus (Biancardi et al. 2016; Ogawa et al. 2011; Wang et al. 2009).

The relationship between systemic inflammation and HRV is important in a wider context as it is a measure of suppressed vagal nerve activity and a dysfunctional cholinergic anti-inflammatory reflex (reviewed (Huston and Tracey 2011)) and hence is accepted as a surrogate marker of global autonomic dysfunction as the vagus nerve acts on both the cardiac sinoatrial node and the reticulo-endothelial system (Borovikova et al. 2000) (reviewed (Herlitz et al. 2015)). It is also of interest that given the existence of data demonstrating that the extent of HRV suppression correlates with the levels of systemic inflammation (Durosier et al. 2015; Herry et al. 2016) (review (Cooper et al. 2015)), that HRV parameters could act as surrogate markers of systemic inflammation, which could potentially prove to be objective biomarkers for CFS patients in the early stages of their illness (Durosier et al. 2015). However, low HRV values are also seen in patients suffering from major depressive disorder (Brunoni et al. 2013), possibly as a result of associated high levels of peripheral PICs (reviewed (Berk et al. 2013)) and hence HRV values are unlikely to be able to differentiate patients with CFS from those with a similar symptom presentation as a result of major depressive disorder.

### Development of neuroinflammation, neurocognitive and neuroimaging abnormalities

#### Development of neuroinflammation

There is accumulating data demonstrating a causative association between the development of chronic systemic inflammation and disruptions in resting state connectivity, the development of GM and WM atrophy and lesions, and reduced cerebral perfusion (Adam et al. 2013; Felger et al. 2015; Labrenz et al. 2016; Lekander et al. 2016; Marsland et al. 2015; Riverol et al. 2012; Sankowski et al. 2015; Sonneville et al. 2013). There is also a large and increasing body of evidence indicating a causative relationship between the presence of chronic systemic inflammation and the existence of cognitive disability in patients diagnosed with a range of neuroinflammatory, neurodegenerative and neuroprogressive conditions (Gorelick 2010; Marsland et al. 2015; Sartori et al. 2012).

Inflammatory signals can reach the brain via humoral and neural routes to activate the HPA axis (Morris and Berk 2015). The humoral route involves direct or indirect cytokine signalling, either through direct access to the brain via regions where the integrity of the blood-brain barrier (BBB) is compromised or absent, such as the choroid plexus or other circumventricular organs (CVOs) (Morris et al. 2013), or by direct entry via saturable BBB transport systems or an indirect induction of cytokines and other inflammatory mediators, such as prostaglandins, and their subsequent release into the CNS parenchyma or via provocation of an increase in BBB permeability (Morris et al. 2015b; Seruga et al. 2008). The neural route involves direct stimulatory action of PICs on peripheral afferent neurons of the vagus nerve (Goehler et al. 2000; Johnston and Webster 2009).

Entry of PICs into the brain can have profound pathological consequences either directly or indirectly. There is now overwhelming evidence that transduced inflammatory signals provoke the development of chronic neuroinflammation secondary to the sequential activation of microglia and astrocytes. Activated microglia secrete a range of neurotoxic molecules such as tumour necrosis factor (TNF)-α, IL-6, IL-1β, ROS, RNS, COX-2, prostaglandin E_2_ (PGE_2_), glutamate and, in some cases, quinolinic acid (Morris et al. 2015b; Morris and Maes 2013b). Moreover, the release of PICs can in themselves act as independent sources of RNS, primarily NO, and other neurotoxins via their capacity to upregulate iNOS, COX-2 and PGE_2_ (Morris et al. 2015a; Sofroniew and Vinters 2010). Unsurprisingly, the production of these neurotoxins can exert profound and detrimental effects on neurotransmitter systems, and neural integrity and function (Morris et al. 2015b).

Activated microglia and subsequent release of PICs and glutamate, combined with reduced glutamate reuptake by activated astrocytes, can lead to the development of glutamate neurotoxicity with resulting damage to glutamatergic neurones and disruption to glutamatergic neurotransmission (Noda 2016; Robel et al. 2015; Takeuchi et al. 2006), while the I&OS generated by such activation damage A9 dopamine and A6 noradrenaline neurones thereby respectively disrupting dopaminergic and noradrenergic neurotransmission (Nagatsu and Sawada 2006; Tripathy et al. 2015). There is also an accumulating body of evidence indicating that elevated ROS and RNS in the CNS can inhibit dopaminergic neurotransmission by inhibiting dopamine receptors (Morris et al. 2017d). Elevated CNS PIC levels, and subsequent activation of the p38 mitogen-activated protein kinase (MAPK) signalling system, can also adversely affect the synthesis, reuptake and release of serotonin (Miller et al. 2013). Elevated PICs in the CNS also provoke the activation of the tryptophan catabolite pathway, depleting levels of tryptophan, which is the precursor of 5-HT, as well as creating another dimension of neuropathology via the synthesis of several neurotoxic tryptophan catabolites including the potentially neurotoxic quinolinic acid (Miller et al. 2013) (reviewed (Morris et al. 2016e)).

There are also some data to suggest that activated microglia inhibit GABAergic neurotransmission, but perhaps counterintuitively this action would appear to have a neuroprotective effect (Chen et al. 2014). It is also noteworthy that this would appear to be contrary to the effects of systemic inflammation on the GABA system, leading to its activation, which is considered to be one source of apparently idiopathic chronic pain (Jang et al. 2017). This may be a mechanism underpinning the presence of the chronic intractable pain reported by many CFS patients (Nijs et al. 2012). In this context it is also worth noting that microglial activation, most notably in the basal ganglia, is also a well-documented cause of ‘unexplained’ chronic pain (Jeon et al. 2017).

Systemic LPS can enter the CNS via regions of BBB permeability such as the circumventricular organs and area postrema, in much the same way as PICs, and engage TLR4 receptors on microglia leading to their activation (Hines et al. 2013; Konsman et al. 2002; Sandiego et al. 2015). The resulting neurocognitive dysfunction has much the same origins as microglial activation induced by PICs but there is some evidence to suggest that the adverse effects on adult neurogenesis, memory deposition and recall, synaptic plasticity and long-term potentiation following elevated systemic LPS is primarily mediated by elevated IL-1β in the hippocampus (Abareshi et al. 2016; Li et al. 2017; Nolan et al. 2005).

#### Chronic neuroinflammation as the cause of neurocognitive and neuroimaging abnormalities

It should also be stressed at this junction that microglia and astrocytes play indispensable roles in maintaining CNS homeostasis in areas such as synaptic plasticity and long-term potentiation, which are essential for the deposition and retrieval of memory representations, together with oxygen and nutrient delivery to neurones; thus dysregulated activity of these glial cells is detrimental to cognitive function (Sofroniew and Vinters 2010; Xavier et al. 2014). Hence the development of functional gliopathology in combination with disturbances to neurotransmission following the activation of microglia and astrocytes discussed above could explain, at least in part, the multiple lines of evidence demonstrating cognitive dysfunction in patients with CFS.

This would also seem to be true of neuroimaging abnormalities seen in CFS patients as there is direct evidence that neuroinflammation resulting from microglial activation disrupts resting state functional connectivity (Colasanti et al. 2016) and there is copious evidence that neuroinflammation is a cause of structural damage and of GM and WM atrophy in a range of neurological and medical conditions (Calabrese et al. 2015; Chen et al. 2015; Cheriyan et al. 2012; Chiang et al. 2017; Raj et al. 2017; Tóth et al. 2017; Zhang et al. 2016).

The existence of chronic ONS in the CNS following the activation of microglia and astrocytes subsequent to the existence of chronic peripheral inflammation may also explain the development of cerebral hypoperfusion in CFS patients. Briefly, high levels of NO and ROS result in oxidative damage to lipids, proteins and DNA in the endothelial cells of the BBB resulting in a pattern of escalating damage to such cells and a concomitant loss of the cytoprotective effects of NO normally derived from endothelial nitric oxide synthase (eNOS) (Lucas et al. 2015; Morris and Maes 2014). This is the result of oxidative inactivation of tetrahydrobiopterin (BH_4_), which is one of the enzyme’s essential cofactors, and changes in levels of arginine and calcium ions (Burghardt et al. 2013; Mitchell et al. 2007; Montezano and Touyz 2012). It should also be noted at this juncture that chronic peripheral inflammation can also impair endothelial eNOS function (Burghardt et al. 2013).

The mechanism underpinning depleted BH_4_ levels under such conditions involves ROS-induced oxidation of BH_4_ to dihydrobiopterin (BH_2_), subsequently reducing levels of the former molecule in the endothelium of the BBB (Najjar et al. 2013). The subsequent decrease in the BH_4_ to BH_2_ ratio results in the inhibition of eNOS while simultaneously uncoupling arginine as its substrate thereby enabling engagement with environmental oxygen and increased production of superoxide ions (Bouloumie et al. 1999; Moens and Kass 2006; Najjar et al. 2013). The resultant combination of superoxide ions with NO results in further increases in levels of ONOO^−^, thereby inducing increased oxidation of BH_4_ to BH_2_, further decreasing the activity of eNOS in an escalating positive feedback loop (Chen et al. 2010; Szabó et al. 2007).

Crucially, reduced eNOS activity can deplete endothelial NO levels, ultimately resulting in significantly impaired CBF (Najjar et al. 2013; Toda and Okamura 2012). The development of this phenomenon also appears to be associated with impaired vasodilation which also stems from impaired neurovascular eNOS-dependent synthesis of NO (Li et al. 2016a; Liu et al. 2016; Najjar et al. 2013). Furthermore, persistent cerebral hypoperfusion can compromise endothelial mitochondrial respiration further increasing the formation of ROS in BBB endothelial cells (Aliev et al. 2010, 2014; Liu and Zhang 2012), which in turn promotes increased eNOS uncoupling, further lowering endothelial NO levels, and a pattern of incrementally decreasing cerebral perfusion in a positive feedback loop (Antoniades 2006; Chen et al. 2010; Lavoie et al. 2010).

Importantly, the existence of positive feedback loops in the brain and periphery, such as those discussed above, can lead to a pattern of increasing I&ONS, which can trigger a state of metabolic and immune downregulation potentially accounting for a range of apparently conflicting data reported by researchers investigating these domains even in participants afforded a diagnosis of CFS according to the international consensus criteria. These processes form the focus of the remainder of this paper and are discussed below.

### Increasing I&ONS and the switch to immune and metabolic downregulation

#### Advent of hypernitrosylation

Reversible protein *S*-nitrosylation, denitrosylation and transnitosylation of protein cysteine thiols effects the vast bulk of NO cellular signalling and enables the homeostatic regulation of virtually every dimension of redox-dependent protein signalling, largely determining protein function, stability and trafficking (Banerjee 2012; Hill and Bhatnagar 2012; Paulsen and Carroll 2010; Winterbourn and Hampton 2008).

From the perspective of this paper, the key point to stress is that this NO-induced post-translational modification plays an indispensable role in maintaining cellular homeostasis in the face of increasing levels of oxidative stress (Gorelenkova Miller and Mieyal 2015; Okamoto and Lipton 2015). In such an environment, moderate increases in levels of ROS and NO lead to a defensive pattern of increased *S*-nitrosylation of crucial structural and functional proteins as a shield against irreversible oxidation of cysteine thiols and subsequent prolonged or even permanent changes in their secondary and tertiary conformation leading to inactivity and/or immunogenicity (Kohr et al. 2014; Penna et al. 2014; Sun and Murphy 2010; Sun et al. 2006). However, further increases in O&NS lead to impaired activity of denitrosylases such as the thioredoxin system, *S*-nitrosogluthathione reductase, protein disulphide isomerase, superoxide dismutase and glutathione peroxidase, which maintain the reversibility of *S*-nitrosylation leading to a state of protracted or even irreversible nitrosylation which has been described as hypernitrosylation (Wu et al. 2010; Wu et al. 2011) (reviewed (Morris et al. 2017c)).

A state of increased nitrosylation in CFS is indicated by findings that IgM responses to NO-tryptophan, NO-tyrosine, NO-albumin and NO-cysteinyl are increased in CFS (Maes et al. 2006). Interestingly, in CFS, increased bacterial translocation is associated with indicants of increased nitrosylation (Maes and Leunis 2014). A wider discussion of the potential role of NO and ONOO^−^ in the pathogenesis and pathophysiology of CFS may be found in a recent review by Monro and Puri (2018). This is of importance as the development of ‘hypernitrosylation’ can provoke a switch from an inflammatory environment with excessive activation of immune pathways to an environment of hypo-inflammation, impaired energy production and metabolic downregulation, which may be exacerbated in individuals with high levels of translocated LPS as high systemic concentrations of this antigen may result in the same ultimate endpoints (Morris et al. 2016b, c). This will form the theme of the remainder of this paper with initial impairment in oxidative phosphorylation and mitochondrial dynamics considered immediately below.

### Hypernitrosylation and impaired mitochondrial performance

There is a considerable and accumulating body of data demonstrating that *S*-nitrosylation of key mitochondrial enzymes and structural proteins plays an indispensable role in the redox-based regulation of mitochondrial respiration and other aspects of energy production (Doulias et al. 2013; Mailloux et al. 2014). In sum, readily reversible *S*-nitrosylation negatively regulates the function of a myriad of proteins involved in oxidative phosphorylation, the tricarboxylic cycle, gluconeogenesis, glycolysis, generation of mitochondrial ROS, mitochondrial permeability transition, as well as apoptosis (Doulias et al. 2013; Mailloux et al. 2014). Hence hypernitrosylation has the capacity potentially to impair cellular energy generation and, in particular, to lead to inhibitory nitrosylation of crucial cysteine thiols of enzymes in the electron transport chain (ETC) such as complex I (Drose et al. 2014; Murray et al. 2012; Piantadosi 2012), cytochrome *c* oxidase (complex IV) and, to a lesser extent, complex II (Sarti et al. 2012a; Zhang et al. 2005). Unsurprisingly, such inhibition impairs oxidative phosphorylation and the production of ATP, and depletes levels of mitochondrial glutathione owing to increased production of ROS by a dysfunctional ETC (Sarti et al. 2000, 2003a, b, 2012b; Zhang et al. 2005). There are also data demonstrating that prolonged inhibition of cytochrome *c* oxidase increases ATP production by glycolysis at least in some cell types as an attempt to mitigate against cell death via apoptosis or necrosis (Almeida et al. 2001; Bolanos et al. 2010; Burwell et al. 2006; Shiva et al. 2007; Sun et al. 2007).

In addition, prolonged nitrosylation of the mitochondrial regulatory proteins PINK-1, PARKIN and DRP-1 compromises mitochondrial dynamics as a result of impaired mitophagy, increased degeneration and increased fission (Nakamura et al. 2010; Oh et al. 2017; Ozawa et al. 2013; Reddy et al. 2011). This may be relevant from the perspective of activity intolerance reported by many CFS patients as intact mitochondrial dynamics enables the adaptation of an individual to continuous or incremental exercise regimes (Trewin et al. 2018; Yan et al. 2012).

### Hypernitrosylation and the development of immune suppression and cellular hibernation

**Fig. 1:**
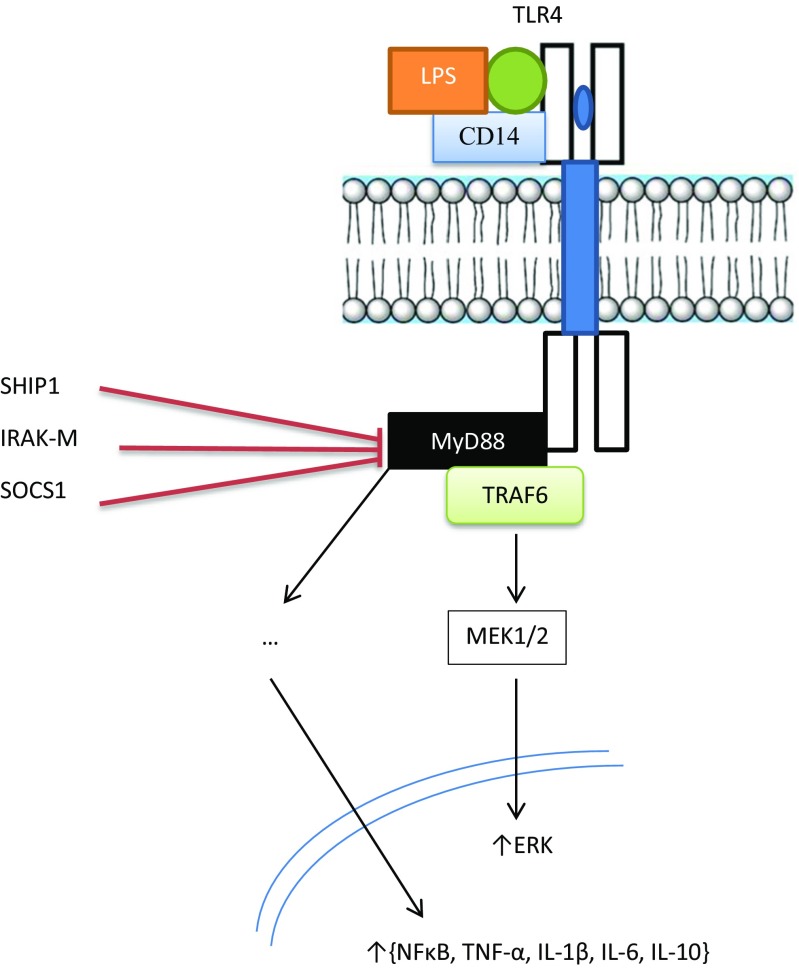
Acute stimulation of TLR4 leads to the recruitment of MyD88, MyD88-adaptor-like (MAL), IL-1 receptor-associated kinase-4 (IRAK-4), toll/IL-1 receptor (TIR)-domain-containing adaptor-inducing IFNβ (TRIF) and TRIF-related adaptor molecule (TRAM), ultimately provoking the initiation of signalling cascades that converge to activate NFκB, MAPKs and IFN response factors (IRFs) with the subsequent production of inflammatory mediators such as type 1 interferons and PICs. However, in a state of endotoxin tolerance the TLR4 response is reprogrammed via the upregulation of the inhibitory proteins SHIP1, suppressor of cytokine signalling 1 (SOCS1) and the pseudokinase IRAK-M leading to the production of IL-10 and TGF-β1 leading to a downregulated and anti-inflammatory immune response

Prolonged or intractable *S*-nitrosylation characteristic of hypernitrosylation may lead to changes in the activity of proteins and signalling pathways regulating inflammation and energy production, leading to a state of hypo-inflammation and metabolic downregulation. For example, hypernitrosylation of a crucial cysteine residue of p50 silences NF-κB-dependent gene transcription via several different mechanisms (Bogdan 2001; DelaTorre et al. 1997, 1998, 1999; Kelleher et al. 2007). In addition, hypernitrosylation of cytosolic enzyme inhibitory kappaB kinase beta impairs its levels of phosphorylation (Reynaert et al. 2004) leading to diminished proteasomal degradation of inhibitory kappaB which maintains NF-κB in an inactive state (Hess et al. 2005; Marshall et al. 2004; Reynaert et al. 2004). In addition, hypernitrosylation can further contribute to the development of immune suppression or an hypo-inflammatory state by the downregulation of TLR signalling via inhibition of MyD88 thereby inhibiting the immune response to acute pathogen invasion (Into et al. 2008). Increased NO levels may also upregulate *IRAK-M* mRNA and protein expression of interleukin receptor associated kinase-M (IRAK-M), which is a key negative regulator of TLR signalling although there is no compelling data indicating that this process is enabled by *S*-nitrosylation either directly or indirectly (del Fresno et al. 2004; Gonzalez-Leon et al. 2006). These pathways are illustrated in Fig. 1.

**Fig. 2:**
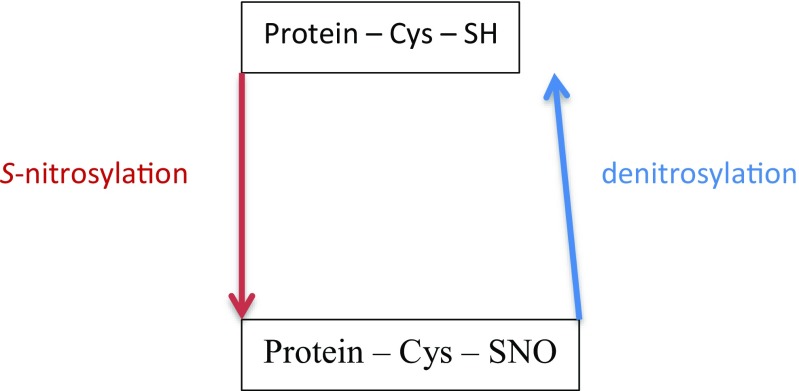
Proteins undergo reversible *S*-nitrosylation and denitrosylation initiated by the covalent addition and release of NO probably derived from N_2_O_3_ in a hydrophobic environment. Denitrosylation may be mediated by several molecular players such as GSNOR (*S*-nitrosoglutathione (GSNO) reductase) and the thioredoxin system. Transnitrosylation is another major route mediating protein nitrosylation. Transnitrosylation of GSH leading to the formation of native protein and GSNO is a major element enabling this process. Reformation of GSH by NADPH involves NADH-dependent reduction by GSNOR to generate GSH. The GSH and thioredoxin systems are progressively inactivated in an environment of increasing chronic ONS leading to the inhibition of denitrosylation and transnitrosylation and a state of protein ‘hypernitrosylation’

Prolonged *S*-nitrosylation (see Fig. 2) can also lead to profound metabolic changes via upregulation of the protein subunit hypoxia-inducible factor-1α (HIF-1α), through upregulation of *HIF1A* and/or stabilisation of this subunit, under non-hypoxic conditions (Kasuno et al. 2004; Li et al. 2007; Yasinska and Sumbayev 2003) and activation of phosphatidylinositol 3-kinase (PI3K)/protein kinase B (Akt)/mTOR signalling (Gupta et al. 2017; Kwak et al. 2010; Lopez-Rivera et al. 2014; Numajiri et al. 2011). There is also evidence that mTOR is directly activated as a result of inhibitory *S*-nitrosylation of tuberous sclerosis complex 2 (TSC2), an inhibitor of mTOR (Lopez-Rivera et al. 2014) and nitrosylation-mediated activation of Ras, a small GTPase which is a positive regulator of mTOR (Lee and Choy 2013). Prolonged nitrosylation may also provoke changes in metabolic pathways via the upregulation of GSK-3 (Morris et al. 2017c). Furthermore, activated GSK-3 and PI3K/Akt signalling in tandem or separately can provoke metabolic and bioenergetic dysregulation by inhibiting the enzyme 5′-adenosine monophosphate (AMP)-activated protein kinase (AMPK) (Park et al. 2014; Suzuki et al. 2013).

These observations may be directly relevant as far as the pathogenesis of CFS is concerned as activation of HIF-1α, PI3K/Akt/mTOR and GSK-3 signalling pathways and inhibition of AMPK, in the context of decreased canonical NF-κB activation, are major elements in the development of a phenomenon described as endotoxin tolerance which involves profound systemic immune and metabolic downregulation, with the latter phenomenon involving sequential shifts in energy production via glycolysis and oxidation of fatty acids, following prolonged TLR upregulation in macrophages and monocytes (Liu et al. 2011a, 2012). This phenomenon is mediated, at least in part, by epigenetic reprogramming ‘masterminded’ by sirtuin 1 (SIRT1) (Liu et al. 2011a, 2012) and in this context decreased levels of canonical NF-κB signalling induced by persistent nitrosylation would be expected to activate SIRT1 (Kauppinen et al. 2013; Vaziri et al. 2001; Yang et al. 2012). This is important as SIRT1 nitrosylation in the context of high levels of classical NF-κB signalling secondary to chronically elevated I&ONS or the response to acute pathogen invasion leads to its inactivation (Kalous et al. 2016; Nakazawa et al. 2017; Shinozaki et al. 2014).

Before moving on to consider the mechanisms underpinning the development of endotoxin tolerance, it is important to note that this phenomenon resolves within days and sometimes weeks in the context of DAMP- or pathogen-associated molecular pattern-mediated engagement of PRRs, but in the context of the same result mediated by chronically elevated I&ONS and hypernitrosylation a state similar to endotoxin tolerance could be protracted or even permanent in the absence of ameliorative interventions.

### Mechanisms underpinning metabolic downregulation in endotoxin tolerance

During the initial inflammatory phase following TLR activation, NF-κB upregulates the Akt-mTOR-HIF-1α pathway, leading to a surge in ATP production via aerobic glycolysis (Cheng et al. 2014a; Srivastava and Mannam 2015; van Uden et al. 2008). Such upregulation leads to inhibition of the tricarboxylic acid (TCA) cycle and increased production of mitochondrial ROS, leading to a fall in mitochondrial ATP production, mitochondrial structural damage, increased mitophagy and a switch in cytochrome oxidase subunits designed to increase the efficiency of the ETC (Cheng et al. 2014a; Semenza 2011; Zhong et al. 2010). Inhibition of the TCA cycle and oxidative phosphorylation is mediated by increase in the transcription of pyruvate dehydrogenase kinase with the subsequent inhibition of pyruvate dehydrogenase and conversion of pyruvate into acetyl coenzyme A (acetyl-CoA). This inhibition is accompanied by a concomitant HIF-1α-mediated increase in the conversion of pyruvate to lactate with the resultant production of ATP and NAD^+^ by aerobic glycolysis (Kim et al. 2006).

The continuation of ATP production and NAD^+^ by this route is enabled by HIF-1α-induced upregulation of lactate dehydrogenase (Semenza et al. 1996). The switch from ATP generation from oxidative phosphorylation instigated by the upregulation of the transcription factor is further enabled by increased expression of glucose transporters and glycolytic enzymes (Hanahan and Weinberg 2011; Semenza 2003). The importance of Akt-mTOR-HIF-1α signalling in the development of endotoxin tolerance is emphasised by data demonstrating that inhibition of this pathway prevents the development of immune and metabolic downregulation in macrophages and monocytes (Cheng et al. 2014a).

However, this rapid burst of ATP production and concomitant increases in NADH production enabled by activation of the of Akt-mTOR-HIF-1α signalling system is a short-lived phenomenon and the subsequent fall in ATP and NADPH generation leads to a relative rise in AMP and NAD^+^ (Adriouch et al. 2007; Haag et al. 2007), leading to the activation of AMPK (Gómez et al. 2015) and the sequential activation of the NAD^+^-sensitive SIRT family members sirtuins 1, 6 and 3 (Liu et al. 2011a, 2012, 2015a). Once activated, these SIRTs play the dominant role in the development of a hypo-inflammatory state and an environment of metabolic downregulation and reductions in glycolysis and TCA-induced oxidative phosphorylation characterised by mitochondrial ATP production via fatty acid oxidation (Gómez et al. 2017; Vachharajani et al. 2016). This adaptive low-energy state is often described as cellular ‘hibernation’ (Levy et al. 2005; Liu et al. 2011a; Singer 2008) (reviewed (Singer 2017)) and it is important to stress that this phenomenon is not limited to macrophages and monocytes but also takes place in hepatocytes and striated muscle cells (Carré and Singer 2008; McCall et al. 2011; Singer 2007, 2008).

Once activated, SIRT1 upregulates the transcription of peroxisome proliferator-activated receptor γ coactivator-1 alpha with a subsequent and concomitant increase in mitochondrial biogenesis and respiration mediated by fatty acid oxidation. The mechanisms underpinning this process include increased cellular uptake of fatty acids via the CD36 membrane transporter and increased transfer of these molecules into mitochondria via upregulation of several enzymes including palmitoyltransferase I, which governs the rate of fatty acid oxidation (Liu et al. 2012; Vachharajani et al. 2014; Wanders et al. 2010) (reviewed (Qu et al. 2016)). SIRT1 also exerts a range of protective effects on mitochondria aimed at maximising energy generation and fostering cellular and organelle survival. These effects include: upregulating cellular anti-oxidant defences, via activation of nuclear factor (erythroid-derived 2)-like 2; increasing mitophagy to remove damaged mitochondria; and maintaining mitochondrial membrane potential, thereby inhibiting the development of mitochondrial permeability transition pore opening (Price et al. 2012; Song et al. 2017).

SIRT1-induced upregulation of SIRT6 also encourages the switch from ATP generation via aerobic glycolysis to ATP generation via fatty acid oxidation, via direct and indirect inhibition of glycolysis, increased fatty acid oxidation and a range of effects broadly encouraging mitochondrial survival in a low-energy state (Cheng et al. 2016; Sebastian et al. 2012). Direct inhibitory effects of SIRT6 on glycolysis include downregulation of glucose transporter 1 and inhibition of lactate production (Elhanati et al. 2016; Long et al. 2017), while indirect effects stem from inhibition of HIF-1α (Sebastian et al. 2012). SIRT6 elevation also preserves mitochondrial membrane potential, preventing mitochondrial permeability transition pore (mPTP) opening in a similar manner to SIRT1 (Cheng et al. 2016).

**Fig. 3:**
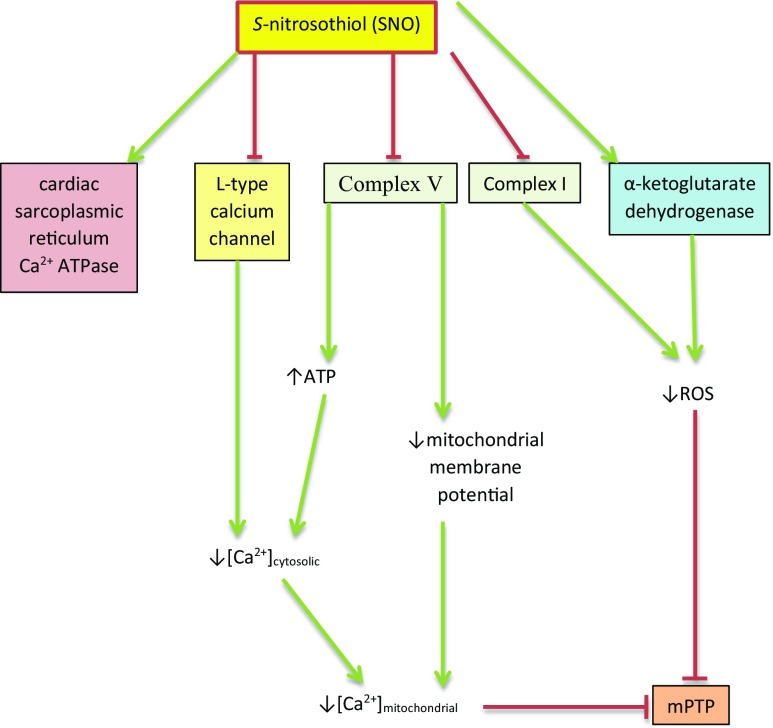
*S-*Nitrosylation leads to the inhibition of Complex 1, Complex IV, the F1F0ATPase (Complex V) and possibly Complex II leading to the reduction of ATP production and a decrease in ROS production with a subsequent increase in production of ATP via aerobic glycolysis. *S-*Nitrosylation can also compromise mitochondrial function while increasing the survival of the organelle by inhibiting uptake of calcium ions and reduction of cytosolic calcium ions via inhibition of SERCA, further reducing mitochondrial ATP and ROS production. *S-*Nitrosylation also inhibits key enzymes of the TCA cycle, such as aconitase and α-ketoglutarate dehydrogenase, and regulates those involved in fatty acid metabolism, thus further inhibiting oxidative phosphorylation and stimulating aerobic glycolysis and mitochondrial fatty acid oxidation

Upregulation of SIRT3, an NAD^+^-dependent mitochondrial protein, also deacetylates and activates mitochondrial enzymes involved in fatty acid β-oxidation, amino acid metabolism, the TCA cycle, the ETC and antioxidant defences leading to increased mitochondrial ATP production (Ahn et al. 2008; Ansari et al. 2017). In addition, increased SIRT3 activity inhibits mitochondrial ROS production and the activity of several components of the mPTP, thereby encouraging mitochondrial survival (Kincaid and Bossy-Wetzel 2013; Tseng et al. 2013) (see Fig. 3).

It is also noteworthy that the sequential activation of these SIRTs corresponds with the inactivation of AMPK (Jiang et al. 2014; Liu et al. 2015b). Unsurprisingly, the causes of this phenomenon have been the subject of intensive research, and while the process may be multifactorial in origin, the weight of data implicates inhibition by activated GSK-3 (Park et al. 2014; Suzuki et al. 2013). The upregulation of GSK-3 in turn appears to result from the termination of the inhibitory influence of Akt/mTOR signalling by the upregulation of SIRT1 and SIRT6 and the termination of ATP production by aerobic glycolysis (Frost and Lang 2011; Ghosh et al. 2010; Hermida et al. 2017; Pillai et al. 2014). Another contributing factor underpinning the inactivation of AMPK might be the relative increase in mitochondrial ATP production and a reduction in mitochondrial ROS production as a result of a switch to fatty acid β-oxidation from aerobic glycolysis (reviewed (Nsiah-Sefaa and McKenzie 2016)) would be below the cellular AMP and ROS thresholds expected to trigger the activation of the enzyme (Morris et al. 2017d).

### SIRT1 activation and subsequent immune downregulation

Once activated, SIRT1 also plays a pivotal role in the development of a hypo-inflammatory immune response. In essence, this is achieved by inhibiting the transcription of IL-1β, TNF-α and other proinflammatory genes and suppressing inflammatory responses by deacetylating and inhibiting the incumbent p65 component of the NF-κB complex while inducing the expression of RelB (Kauppinen et al. 2013; Liu et al. 2011a; Yang et al. 2012) as well as causing gene-specific regulation as histone modifiers (Foster et al. 2007; Gazzar et al. 2008; Liu et al. 2015a; McCall et al. 2010; Yoza et al. 2006).

From a more mechanistic perspective, SIRT1 deacetylates histone protein H1K27 and lysine 310 (Lys310) of RelA/p65 and its continued binding recruits RelB to promoters of target anti-inflammatory genes (Liu et al. 2011a; Millet et al. 2013). In this instance RelB acts as a dual-function transcription factor; it increases silent facultative chromatin at promoters of pro-inflammatory genes via direct interaction with histone H3 lysine 9 methyltransferase G9a, heterochromatin protein 1, assembly of high mobility group box 1 and DNA CpG methylation; (Chen et al. 2009; Gazzar et al. 2009; Millet et al. 2013; Yoza and McCall 2011) and active euchromatin at anti-inflammatory genes via association with the NF-κB subunit p50 and the resultant transcription of nuclear factor of kappa light polypeptide gene enhancer in B-cells inhibitor, alpha (McCall et al. 2010). In addition, SIRT6 also deacetylates RelA/p65 and histone H3K9Ac to exert additional anti-inflammatory activity (Gil et al. 2013; Kawahara et al. 2009).

However, the activation of SIRT 1 and SIRT 6 are not the only elements involved in the immune downregulation characteristic of endotoxin tolerance as miRNA activity, soluble factors such as IL-10 and TGF-β1 and the concomitant downregulation of proteins enabling TLR signalling and upregulation of proteins such as SH2 (Src Homology 2)-containing inositol phosphatase (SHIP) and IRAK-M, known to inhibit TLR signalling, all play an indispensable role (reviewed (Wisnik et al. 2017; Fu et al. 2012)). Crucially, these processes may begin with the activation of indoleamine 2,3-dioxygenase (IDO) which is activated in an environment of excessive I&OS (Morris et al. 2016a), as discussed below.

## I&ONS and the development of endotoxin tolerance via IDO upregulation

Chronic I&ONS can also provoke the development of endotoxin tolerance by inducing the transcriptional activation of IDO (Kim et al. 2015c; Wichers and Maes 2004) leading to upregulation of the kynurenine pathway, aryl hydrocarbon receptor (AhR) activity and increased levels of TGF-β1 (Bessede et al. 2014; Wirthgen and Hoeflich 2015) and IL-10 (Alexeev et al. 2016; Lanis et al. 2017) via well documented mechanisms (reviewed (Wirthgen and Hoeflich 2015)).

The upregulation of AhR activity is of interest given data presented in the previous section as increased activity of this cytosolic transcription factor leads to upregulation of RelB and non-canonical NF-κB signalling (Salazar et al. 2017; Vogel et al. 2013). Mechanistically, these effects appear to be mediated by transcriptional upregulation of RelB (de Souza et al. 2014; Thatcher et al. 2007) and subsequent physical engagement between RelB and AhR to produce dimers capable of modulating the expression of NF-κB-sensitive genes (Vogel et al. 2008). AhR-upregulated RelB also stimulates and maintains the transcription of miR-146a (Zago et al. 2014, 2017). This is of importance as miR-146a is a dominant player in the development and maintenance of the hypo-inflammatory environment characteristic of endotoxin tolerance (Banerjee et al. 2013; Nahid et al. 2009). Mechanistically, this inhibitory effect is enabled by suppressing TLR signalling pathways by reducing the translation of *TNF receptor associated factor 6* (*TRAF6*), *interleukin-1 receptor-associated kinase 1* (*IRAK1*), *IRAK2* and *interferon regulatory factor 3* (*IRF3*), which are positive adaptor kinases of MyD88-mediated signalling and hence their inactivation results in reduced activity of both NF-κB and IRF3 (Nahid et al. 2011) (reviewed (Testa et al. 2017)).

The upregulation of TGF-β1 also results in upregulated non-canonical NF-κB signalling (Pallotta et al. 2011; Shi and Massague 2003). Increased activation of this cytokine also upregulates pseudokinase IRAK-M (Pan et al. 2010; Srivastav et al. 2015; Standiford et al. 2011). This is significant because IRAK-M would appear to be the ‘master regulator’ of the TLR pathway suppression characteristic of the state of endotoxin tolerance in PMBCs (del Fresno et al. 2007; Escoll et al. 2003; Stiehm et al. 2013; van’t Veer et al. 2007; Wiersinga et al. 2009). Indeed, the weight of evidence suggests that increased activity of this enzyme alone is sufficient to maintain an LPS-induced hypo-inflammatory state in human macrophages and monocytes (van’t Veer et al. 2007). This is unsurprising given that this molecule can inhibit TLR signalling at multiple levels. TGF-β1 has been established as an indispensable element in the development of endotoxin tolerance-associated SHIP upregulation (Sly et al. 2004; Yang et al. 2015). This may be of particular relevance from the perspective of a putative explanatory model of CFS aetiology as elevated levels of this cytokine in PMBCs and whole blood are a common finding in patients diagnosed according to narrow international consensus criteria and correlate with the severity of a range of symptoms (Blundell et al. 2015; Wyller et al. 2017). Once again, it is noteworthy that this phenomenon is not observed in patients diagnosed according to broader schema which are not internationally recognised such as the ‘alternative CDC criteria’ (Clark et al. 2017).

Upregulated IL-10 also exerts negative effects on TLR signalling by increasing the ubiquination and proteasome-mediated degradation of a range of MyD88-dependent signalling effector molecules such as IRAK-4 and TRAF6 ultimately resulting in reduced phosphorylation and activity of inhibitor of kappa B kinase (IKK), p38 and JNK (Chang et al. 2009). IL-10 is produced by monocytes, macrophages, Tregs and Th2-polarised T cells in a state of endotoxin tolerance, and suppresses the CD8 T and CD4 Th1 type cell response making an indispensable contribution to the development of an anti-inflammatory environment (Jiang and Chess 2006; Littman and Rudensky 2010). The indispensable contribution of IL-10 to the development of endotoxin tolerance (Liu et al. 2011b; Quinn et al. 2012) is of importance from the perspective of this paper as the upregulation of this cytokine is a common observation in CFS patients (Roerink et al. 2017; Wong et al. 2015).

It should be noted that once activated, IDO activity can be maintained by two positive feedback mechanisms. First, TGF-β can target its cellular receptor leading to the upregulation of NF-κB-RelB signalling leading to further transcription of IDO (Pallotta et al. 2011; Shi and Massague 2003). Second, IDO-activated AhRs can in turn upregulate the transcription of *IDO1* (the gene that encodes IDO) via genomic and non-genomic routes (Li et al. 2016b; Litzenburger et al. 2014). Hence once activated, IDO upregulation could be protracted or even chronic.

In addition, there is evidence obtained from human studies that chronic or intermittent translocation of LPS into the systemic circulation can induce a state of tolerance and alternative activation in macrophages and monocytes characteristic of endotoxin tolerance via the activation of IDO, kynurenine and the AhR (Banerjee et al. 2013; del Campo et al. 2011; del Fresno et al. 2008; Pena et al. 2011; Wisnik et al. 2017). Given the existence of LPS translocation in CFS, this mechanism could also contribute to the development of a chronic state resembling endotoxin tolerance.

The importance of IDO activation in the development of endotoxin tolerance is further emphasised by data confirming that interactions between the AhR, kynurenine and TGF-β1 are responsible for the polarisation of activated naïve T cells into the Treg phenotype by the presentation of antigen by tolerogenic antigen-presenting cells (Gandhi et al. 2010; Mezrich et al. 2010). Such phenotypic presentations are considered below.

IDO2 is a homologue of IDO (also known as IDO1), being an immunomodulatory enzyme which catalyses L-trytophan; like *IDO1*, *IDO2* is also located on chromosome 8 in humans but *IDO2* is not as widely expressed as *IDO1* and IDO2 has a distinct signalling role (Metz et al. 2007; Cha et al. 2018). B cell *IDO2* expression has recently been identified as being an essential mediator of autoreactive B and T cells in autoimmune responses (Merlo and Mandik-Nayak 2016; Merlo et al. 2016, 2017). It seems likely, therefore, that IDO2 may be found to play an important role in ME/CFS.

## State of leukocytes in endotoxin tolerance

### Neutrophils

There are not a great deal of data regarding these immune cells in a state of endotoxin tolerance but the evidence in existence is typical of that would be expected if such cells were in a state of immune downregulation**.** For example, Parker and fellow workers reported a downregulation of TLR4 receptors, high levels of IL-8 secretion and impaired oxidative burst in neutrophils in endotoxin tolerance (Parker et al. 2005). Impaired rolling, endothelial cell adhesion and migration to sites of infection have also been reported (Alves-Filho et al. 2008; Ogawa et al. 2003). However, there appears to be considerable variation in the migration of neutrophils in this condition as another research team has reported that this ability may be significantly increased (Ariga et al. 2014).

### Monocytes and macrophages

Stimulated macrophages and DCs in a state of endotoxin tolerance display M2 polarisation as evidenced by decreased production of PICs such as IL-12, IL-6 and TNF-α and an increased production of IL-10 and TGF-β1 (Albrecht et al. 2008; del Fresno et al. 2009). The magnitude of such immune downregulation is thrown into stark relief by data demonstrating that levels of TNF-α, IL-1β and IL-6 in activated monocytes in this condition are typically less than 10–20% of levels in stimulated monocytes extracted from healthy controls (Boomer et al. 2014; Rigato and Salomao 2003; Sinistro et al. 2008).

Macrophages and DCs in a state of endotoxin tolerance also display impaired antigen presentation owing to downregulated major histocompatibility complex (MHC) class II molecules such as human leukocyte antigens – antigen D related (HLA-DRs) and class II transactivator and the costimulatory molecule CD86 which may be caused, at least in part, by elevated levels of TGF-β and IL-10 (Biswas and Lopez-Collazo 2009).

DCs in particular appear to be immature and their activation appears to be impaired as evidenced by reduced production of chemokine (C-C motif) ligand 3 (CCL3) and CCL5 (Albrecht et al. 2008), reduced levels of CD80 and CD86 (Cohen et al. 2004) and evidence of reduced numbers (Ishiyama et al. 2006).

Curiously, while there is evidence of reduced numbers of DCs as a whole (Ishiyama et al. 2006), it would appear that the relative proportion of myeloid DCs increases leading to a higher level of antigenic stimulation overall, in turn leading to the differentiation of naïve T cells along the Th2 pathway but producing T lymphocytes with a characteristically reduced expression of IL-2 and interferon gamma (IFNγ) but normal levels of IL-4 and IL-5 (Ishiyama et al. 2006; Lauw et al. 2000).

These tolerogenic DCs also play a major part in inducing CD4 and CD8 T cell anergy, inhibition of T cell effector and memory responses and inducing the activation and generation of Tregs, which are all characteristic of endotoxin tolerance (Domogalla et al. 2017; Raker et al. 2015).

### T cell characteristics in endotoxin tolerance

A protracted state of endotoxin tolerance is characterised by CD4 T cell exhaustion or anergy, impaired CD8 T cell proliferation and an absolute rise in the numbers and suppressor function of Tregs (Cabrera-Perez et al. 2014; Cao et al. 2015; Strother et al. 2016). Anergic or exhausted T cells are unresponsive to antigenic stimulation and express high levels of inhibitory receptors on their surfaces such as CD69, programmed death receptor-1 (PD-1), CD25, IL-7R and T cell membrane protein-3 (Boomer et al. 2011) (reviewed (Boomer et al. 2014)). While upregulation of each receptor plays a part in maintaining T cell anergy, there is accumulating evidence that PD-1 and its ligands play the dominant role and that immunotherapy directed at this receptor complex can restore T cell function in vivo (Araki et al. 2013; Lee et al. 2015). Such T cells in this hypo-inflammatory state also display evidence of repressive histone methylation in T-bet (a T-box transcription factor), GATA-3 and retinoic acid receptor (RAR)-related orphan receptor gamma, which would go some way to explaining their unresponsiveness to antigenic stimulation (Pachot et al. 2005) (reviewed (Carson and Kunkel 2017)). It is noteworthy that the pattern of T cell anergy is also seen in patients with chronic viral infections because of unrelenting antigenic stimulation (Yi et al. 2010). This level of CD4 T cell dysfunction is also associated with reactivation of latent herpes viruses which can add to the antigenic milieu and, combined with translocated LPS, could afford another avenue maintaining a persistent a hypo-inflammatory environment (Laing et al. 2012; Limaye et al. 2008; Ouwendijk et al. 2013).

PD-1 upregulation appears to be the dominant player in the development and maintenance of CD8 T cell anergy and loss of CD8 T cell numbers seen in an environment of endotoxin tolerance although the underlying mechanism may be different in this case and such upregulation appears to be driven by elevated levels of TGF-β1 (Baas et al. 2016; Danahy et al. 2016). The level of proliferative and functional impairment in memory CD8 T cells extends to all modes of activation, with decreased antigen-dependent sensitivity and an impaired capacity to detect the presence of inflammatory mediators in the immediate microenvironment being repeatedly reported, which dramatically reduces their effectiveness in dealing with reinvading pathogens (Duong et al. 2014).

The increased numbers and suppressive capacity of Tregs seen during the development of endotoxin tolerance also play a major role in the development of T cell anergy either via a contact-dependent mechanism or via increased secretion of TGF-β and IL-10 (Cao et al. 2015; Jiang et al. 2012). Finally, and perhaps predictably, the development of endotoxin tolerance is associated with decreased numbers and function of NK cells, with decreased IFNγ production by CD56 NK cells being the most commonly reported functional impairment (Chiche et al. 2011; Forel et al. 2012; Pan et al. 2014).

## Can immune and metabolic abnormalities be explained by endotoxin tolerance?

### Metabolic downregulation

The data presented above relating to metabolic downregulation are potentially important observations from the perspective of CFS pathogenesis and/or pathophysiology as they could explain apparently diverse observations in PMBCs such as increased energy production by glycolysis (Lawson et al. 2016), impaired pyruvate dehydrogenase function (Fluge et al. 2016), lower mitochondrial ATP production and disordered lipid metabolism compared with healthy controls (Germain et al. 2017; Tomas et al. 2017) and a cellular state of apparent metabolic hibernation (Naviaux et al. 2016). There is also evidence that NAD^+^ levels may be elevated in CFS patients (Ciregia et al. 2016; Mikirova et al. 2012; Naviaux et al. 2016). Furthermore, the dysfunction of the TCA cycle in CFS patients reported by (Yamano et al. 2016), inhibition of glycolysis and reduced levels of pyruvate reported by (Armstrong et al. 2015) and inactivated AMPK together with subnormal levels of IL-6 in the striated muscles of CFS patients reported by (Brown et al. 2015) are all abnormalities consistent with a pattern of metabolic downregulation associated with the cellular hibernation associated with endotoxin tolerance. It is also noteworthy that the impaired level of oxidative phosphorylation in PMBCs of CFS patients recently reported by (Tomas et al. 2017) is consistent with mitochondrial ATP production via the β-oxidation of fatty acids. Furthermore, the recently published new model for chronic disease pathogenesis and treatment by Naviaux (2018) is germane; in this model, the pathophysiology of chronic disease is re-framed in terms of metabokine and mitochondrial signalling abnormalities.

There is also evidence that AMPK becomes inactivated during a state of endotoxin tolerance, probably as a result of elevated GSK-3 activity (Jiang et al. 2014; Liu et al. 2015b). This may be significant from the perspective of exercise intolerance seen in ME/CFS as this kinase is responsible for sensing and enabling increased ATP production and maintaining metabolic homeostasis in striated muscle cells in response to increasing demands for energy during exercise (Morales-Alamo and Calbet 2016; Trewin et al. 2018). AMPK signalling also plays an indispensable role in stimulating increased levels of fatty acid oxidation needed to foster the recovery of skeletal muscle function and dynamics in the aftermath of exercise (Egan and Zierath 2013). In the context of a putative explanatory model of the causes of the CFS, it is noteworthy that AMPK appears to be inactive in the striated muscle of at least some patients afforded this diagnosis (Brown et al. 2015). Hence the phenomenon of endotoxin tolerance could go some way to explaining the muscle dysfunction, exercise intolerance and prolonged recovery time characteristic of CFS (reviewed (Gerwyn and Maes 2017)). In addition, the fact that increased levels of NO and ROS generated by mitochondria during incremental endurance training can lead to oxidative inactivation of AMPK (Morales-Alamo and Calbet 2016) could explain the relative failure of long-term incremental training regimes to produce any clinically significant benefits in the short or long term in recent trials involving CFS patients initially diagnosed according to the widest existing criteria (White et al. 2011).

### Immune phenotype associated with endotoxin tolerance

The concept of endotoxin tolerance would go some way to providing an explanation of the apparently contradictory patterns of pro- and anti-inflammatory cytokine production and Th1 and Th2 biases which are common in the literature (Brenu et al. 2011; Hornig et al. 2017; Hornig et al. 2015; Morris and Maes 2013c; Maes et al. 2012b; Milrad et al. 2017; Montoya et al. 2017; Peterson et al. 2015; Russell et al. 2016). In addition, such different immune patterns may be explained by activation of the compensatory anti-inflammatory reflex system in patients with CFS, including Th-2 and Treg responses and induced immune tolerance (Morris and Maes 2012). This system tends to attenuate the primary immune response thus causing differential immune patterns depending on the stage of illness (e.g. acute episode versus chronic stage). Both concepts could also explain the results of large studies suggesting the development of a progressively hypo-inflammatory state in CFS patients with time and/or over the course of their disease both in terms of cytokine production and surface receptors on PMBCs (Hardcastle et al. 2015; Hornig et al. 2015; Montoya et al. 2017; Russell et al. 2016). Readers interested in further consideration of the different cytokine and PMBC receptor distribution patterns reported in CFS patterns are invited to consult reviews by (Morris and Maes 2013b; Morris et al. 2015a).

The existence of a state akin to endotoxin tolerance could also potentially explain a considerable body of data indicating the presence of exhausted or anergic CD4 and CD8 cells in CFS patients (Brenu et al. 2011, 2016; Klimas et al. 1990; Loebel et al. 2014; Prieto et al. 1989; Straus et al. 1993). This is also true of elevated Foxp3^+^ Tregs which appear to have been universally reported by researchers using advanced flow cytometry techniques (Brenu et al. 2011, 2014b; Curriu et al. 2013; Ramos et al. 2016). There have also been conflicting accounts of CD4 and CD8 T cell numbers both in absolute terms and in relation to each other which could conceivably be explained by patients being in different phases of an illness at the time of testing (Gupta and Vayuvegula 1991; Maes et al. 2015; Natelson et al. 2002; Racciatti et al. 2004). In this context, the work of Maes et al. and of Racciatti et al. would appear to be especially worthy of note as these studies were large, containing 139 and 134 patients respectively, and produced diametrically opposite results with the former reporting a reduced CD4/CD8 ratio while the latter reported an elevated CD4/CD8 ratio (Maes et al. 2015; Racciatti et al. 2004).

NK cell hypofunction is the most well documented immune abnormality in CFS patients (Brenu et al. 2016; Fletcher et al. 2010; Natelson et al. 2002) and there is evidence of abnormal metabolic pathways and impaired signalling activity known to be involved in endotoxin tolerance in this cell type extracted from CFS/ME patients both in terms of miRNA activity and impaired extracellular signal-regulated kinase 1/2, mitogen-activated protein kinase 1/2 and p38 signalling (Huth et al. 2016; Petty et al. 2016). It is also noteworthy that while there is a paucity of research examining macrophages, monocytes and neutrophils, those research teams which have investigated these parameters have reported abnormalities. For example, research teams have reported impaired phagocytosis and evidence of immune senescence in monocytes characterised by reduced expression of HLA-DR antigens (Gupta and Vayuvegula 1991; Prieto-Domínguez et al. 2016; Straus et al. 1993) while a recent study revealed a decrease in DCs overall but an increase in myeloid DCs, which is a characteristic of DC populations in a state of endotoxin tolerance as previously discussed (Brenu et al. 2014b). Several abnormalities in neutrophil phenotype and function have also been reported with reduced respiratory burst and phagocytic capacity together with an increased tendency to apoptosis being the most commonly reported findings (Brenu et al. 2010; Bryceson et al. 2006; Harvey et al. 2016; Kennedy et al. 2004). Other evidence of immune downregulation in at least some patients with CFS/ME includes downregulated TLR4 activity (Light et al. 2009, 2013; White et al. 2012) and a pattern of miRNA expression consistent with immune suppression induced by TGF-β1 signalling (Brenu et al. 2014a; Petty et al. 2016).

It is important to note that while large studies invariably report a plethora of immune abnormalities in patients with CFS/ME (Brenu et al. 2011; Hornig et al. 2015, 2017; Maes et al. 2012a, b, 2015; Milrad et al. 2017; Montoya et al. 2017; Peterson et al. 2015; Racciatti et al. 2004; Russell et al. 2016; Tirelli et al. 1993, 1994), this not true of smaller studies where the results are inconsistent even when participants fulfil the criteria for a CFS diagnosis according to international guidelines (reviewed (Natelson et al. 2002)). The reasons for such conflicting results are not entirely clear but the inclusion of individuals with primary depression, retrospective diagnoses and inconsistencies with cytokine assay approaches have all been cited as possible explanations. In addition, there is no evidence of any immune abnormalities in individuals afforded a diagnosis of CFS or ME based on any diagnostic schema other than the Fukuda or Canadian criteria (Blundell et al. 2015).

## Conclusion

In this paper, it has been shown that, while the aetiology of CFS/ME is currently unknown, there is strong evidence of this illness being associated with a wide range of biological abnormalities, most notably in the neuroendocrine, autonomic, neurological, bioenergetic, redox and immunological domains. It has also been seen that epigenetic variation in immune response genes plays a major role in determining the development of DAMPs post-infection, which is pertinent from the perspective of the aetiology of the illness as the production or presence of these molecules can ‘convert’ an acute pathogenic infection into a state of escalating chronic systemic inflammation, which in turn can give rise to many of the reported symptoms and biological abnormalities. It has further been demonstrated in this paper how this relatively simple concept does indeed lead to a novel explanatory model which explains the major biological observations.
